# Individual differences in cognitive reappraisal usage modulate the time course of brain activation during symptom provocation in specific phobia

**DOI:** 10.1186/2045-5380-3-16

**Published:** 2013-08-12

**Authors:** Andrea Hermann, Verena Leutgeb, Wilfried Scharmüller, Dieter Vaitl, Anne Schienle, Rudolf Stark

**Affiliations:** 1 Department of Psychotherapy and Systems Neuroscience, Bender Institute of Neuroimaging, Justus Liebig University, Otto-Behaghel-Strasse 10H, Giessen 35394, Germany; 2 Bender Institute of Neuroimaging, Justus Liebig University, Giessen, Germany; 3 Department of Clinical Psychology, University of Graz, Graz, Austria

**Keywords:** Specific phobia, Emotion regulation, Cognitive reappraisal, vmPFC, Insula, Habituation, Extinction, fMRI, CBT

## Abstract

**Background:**

Extinction learning is proposed to be one key mechanism of action underlying exposure-based cognitive-behavioral therapy (CBT) in specific phobia. Beyond that, cognitive reappraisal, one important strategy to regulate negative emotions, is a crucial component of CBT interventions, but has been disregarded in previous studies investigating neural change processes in specific phobia. The aim of this study was to investigate the association of individual differences in habitual/dispositional cognitive reappraisal usage and the time course of brain activation during phobic stimulation in specific phobia.

**Methods:**

Dental phobic patients and healthy control subjects participated in a functional magnetic resonance imaging (fMRI) study whilst being confronted with phobic, disgust, fear and neutral pictures. Individual differences in cognitive reappraisal usage were assessed via a self-report questionnaire and correlated with activation decreases over the course of time.

**Results:**

Phobic individuals with higher dispositional cognitive reappraisal scores showed a more pronounced activation decline in the right dorsomedial prefrontal cortex (dmPFC) which might be associated with a diminution of explicit cognitive emotion regulation over the course of time. Less decrease of activation in the right ventromedial prefrontal cortex (vmPFC) and the lateral orbitofrontal cortex (lOFC) over time in subjects with higher cognitive reappraisal scores might be related to a stronger automatic regulation of emotions or even emotional relearning. Additionally, phobic subjects compared with healthy controls showed a stronger habituation of the left dmPFC over the course of symptom provocation.

**Conclusions:**

The results of this study show for the first time that individual differences in cognitive reappraisal usage are associated with the time course of brain activation during symptom provocation in specific phobia. Additionally, the present study gives first indications for the importance of considering individual differences in cognitive reappraisal usage in the treatment of specific phobia.

## Background

Dental phobia - one form of specific phobia - is characterized by an excessive fear in response to and a strong avoidance of phobic situations [1] as for instance dental treatment. Regarding the development of dental phobia, individuals with high levels of dental anxiety report distressing dental experiences up to 18 times more often than low-anxious individuals [2,3]. Classical conditioning is considered as one important mechanism underlying the development of phobic fear in response to distressing events [4,5]. Thereby, a former neutral stimulus (e.g. dentist) acquires a negative affective value by being paired with an unconditioned stimulus (e.g. pain during dental treatment) that elicits an unconditioned response (e.g. fear response). Subsequently, the former neutral stimulus (conditioned stimulus) elicits a conditioned response (e.g. fear) that is similar to the unconditioned response, without being paired with the unconditioned stimulus again. Numerous studies have been conducted in recent years trying to elucidate the neural mechanisms underlying the acquisition of fear in healthy individuals as well as the neural basis underlying phobic fear responses (for an overview see [6]). Supporting a learning theory-based perspective on specific phobia, fear conditioning in healthy subjects and symptom provocation in specific phobia seem to be characterized by similar neural correlates including enhanced amygdala, insula, and dorsal anterior cingulate cortex (dACC) activation (for an overview see [6]). In dental phobia, activation has also been found in basal ganglia and prefrontal cortex regions [7,8].

Based on learning theory, the extinction of conditioned responses is supposed to be the central process underlying exposure based treatments in anxiety disorders as for instance specific phobia [9]. Extinction is defined as the repeated confrontation with a conditioned stimulus without subsequent presentation of the unconditioned stimulus, leading to a reduction of conditioned responding over time. Especially the ventromedial prefrontal cortex (vmPFC) has been emphasized as a key structure underlying extinction learning and retrieval (for an overview see [10]). Correspondingly, reduced vmPFC activation has been shown in patients with specific phobias during symptom provocation [11-13]. Studies investigating the effects of cognitive-behavioral therapy (CBT) on neural correlates of symptom provocation in spider phobia indicate reduced activation of the insula and the dACC [14] as well as increased activation of the vmPFC [13] as a result of successful CBT. All in all, these findings demonstrate that fear acquisition and symptom provocation as well as fear extinction and symptom modification via CBT are characterized by similar neural circuits.

Despite the vast literature on the neural correlates of symptom provocation in specific phobia and the few studies on the effects of CBT on these neural correlates, little is known about the mechanisms of action (e.g. habituation, extinction) underlying such processes of change in phobic patients. There is one H2 15O-positron emission tomography study showing habituation of several brain regions including the amygdala and the insula during prolonged exposure to phobic compared with non-phobic stimuli in spider phobic subjects [15]. These results give first evidence for the neural basis underlying habituation or extinction processes in specific phobia. Generally, it has been claimed that fear extinction in humans comprises more than mere passive learning, namely cognitive processes [16], which might additionally influence conditioned responding during extinction. In line with this, cognitive interventions are important components of CBT [17]. Moreover, it has been shown that adding guided threat reappraisal to exposure treatment in specific phobia led to enhanced between-trial habituation [18] as well as decreased return of fear [19].

Cognitive reappraisal is one prominent form of cognitive emotion regulation defined as reinterpreting a stimulus or situation in a way that reduces its emotional impact [20]. Several functional magnetic resonance imaging (fMRI) studies investigating the neural basis of emotion regulation via cognitive reappraisal found enhanced activation of lateral and medial prefrontal cortical control regions accompanied by reduced activation of emotional arousal-related brain structures like the amygdala and the insula during (successful) emotion regulation (for an overview see [21]). One of our own studies investigated cognitive reappraisal of phobic compared with general aversive stimuli in specific phobia [12]. Results of this study show that cognitive down-regulation of emotional responses to phobic pictures led to reduced activation of insula and dACC compared with just looking at the pictures. In addition, down-regulation of phobic compared with general aversive emotional responses was associated with an increased regulation effort and diminished activation of the right rostral ACC (rACC) and the dorsomedial PFC (dmPFC), regions crucially involved in the cognitive regulation of emotions [21]. Altogether, these results indicate that patients with specific phobia exhibit a phobia-specific regulation deficit reflected in a dysfunctional recruitment of rACC and dmPFC.

Beyond that, previous research has shown that individuals vary in their habitual use of cognitive reappraisal (dispositional cognitive reappraisal) as a strategy to regulate emotions. This individual tendency has been shown to be stable in time [20]. A more frequent use of cognitive reappraisal has been related to better interpersonal functioning, enhanced psychological well-being, and reduced depressive symptoms [20]. On the neurobiological level, dispositional cognitive reappraisal has been found to be correlated with reduced insula activation during the anticipation of negative affective stimuli [22], with reduced amygdala and stronger dACC responses while viewing negative emotional faces [23], and with stronger dACC and dorsolateral prefrontal cortex (dlPFC) responses during response inhibition towards negative emotional material (sad vs. happy faces; [24]). Moreover, studies investigating brain structural correlates found an association of dispositional cognitive reappraisal with dACC [25] and vmPFC [26] gray matter volumes. In sum, these numerous findings regarding individual differences in cognitive reappraisal usage indicate a pivotal role of this factor in the modulation of emotional responses.

Despite generally large effect sizes of exposure-based treatments in specific phobia [27], one challenging future task is to figure out possible factors contributing to individual differences in treatment response and return of fear at follow-up. Considering the abovementioned correlates of dispositional cognitive reappraisal, this factor might be a promising candidate for modulating treatment responses in specific phobia. Individuals might differ in the extent they use cognitive reappraisal to regulate their emotions during an exposure session. This might moreover result in different outcomes of exposure therapy at the end of treatment or at follow-up. Previous results on mechanisms of change in CBT have shown that the ‘performance’ during exposure (e.g. rate of habituation, initial fear responding) is not a good predictor for final therapy success [28]. Furthermore, Craske and colleagues assume that the toleration of fear during exposure is far more important to exposure therapy than the reduction of fear. This general ability to manage ones own emotions is also represented in the strategy of cognitive reappraisal. However, until now there are no studies investigating the influence of individual differences in cognitive reappraisal usage on the neural correlates of extinction-related processes in anxiety disorders, as for instance specific phobia. Because it is difficult to conduct typical exposure therapy during scanning, one first approximation is to investigate the time course of brain activation during symptom provocation. The repeated presentation of phobic stimuli without negative consequences (e.g. pain) might be a plausible equivalent to emotional relearning/extinction learning, habituation as well as to a first exposure session in the treatment of specific phobias.

Hence, we investigated a sample of 27 dental phobic patients and 21 healthy control subjects who underwent fMRI during symptom provocation with phobia-specific, generally disgust and fear inducing as well as affectively neutral pictures. The aim of the study was to identify brain structures showing differential activation over time for phobic compared with neutral stimuli depending on the extent of habitual cognitive reappraisal usage in phobic patients. The emotion regulation questionnaire (ERQ, [20]. German version [29]) was used to measure individual differences in habitual cognitive reappraisal usage. A stronger dispositional use of cognitive reappraisal is supposed to lead to enhanced habituation or emotional relearning (e.g. extinction) because these individuals might be better at spontaneously using reappraisal during symptom provocation. This is expected to result in a stronger reduction of activation in brain regions associated with phobia specific processing (amygdala, insula, dACC, basal ganglia). In addition, extinction-related structures as for instance the vmPFC should exhibit less activation decrease over time because individuals with more pronounced habitual reappraisal use are supposed to show stronger emotional relearning respectively extinction learning. Furthermore, cortical control-related areas (rACC, dmPFC, vlPFC, dlPFC) involved in cognitive reappraisal processes are assumed to exhibit a different time course as a function of habitual cognitive reappraisal usage. It is conceivable that subjects high in cognitive reappraisal are characterized by enhanced activation in these areas over time due to sustained cognitive reappraisal of phobic stimuli during the course of symptom provocation. On the other hand, these individuals might be more effective in reducing emotional responses and therefore might show a stronger reduction of activation in these PFC structures over time because regulation might take place faster automatically rather than by explicit and effortful cognitive reappraisal processes. Additionally, we were interested in the specificity of the effects and therefore compared phobic with general affective responses (fear and disgust) as well as phobic individuals with healthy control subjects.

## Methods

### Subjects and procedure

Twenty-seven individuals (15 females/12 males) with dental phobia according to DSM-IV-TR [1] and 21 healthy control subjects (12 females/9 males) participated in a functional magnetic resonance imaging study (mostly overlapping with the sample from [8]). Three further subjects in the phobia group were excluded due to problems in stimulus timing. This study is part of a larger project investigating the functional and structural basis of dental phobia. The main results of this fMRI study have recently been published by [8]. The phobic participants had a mean age of 31 years (SD = 11.56 years) and the healthy controls of 29.81 years (SD = 9.13). They completed the Dental Anxiety Scale (DAS; [30] range: 4-20; cut-off: 15 for highly anxious or phobic individuals), a questionnaire assessing the experienced anxiety during dental treatment. All subjects were right-handed. The participants were recruited via announcements in local newspapers and at the university campus. All subjects gave written informed consent. The local ethics committee of the department 06 of the Justus Liebig University Giessen (Germany) approved the study procedure and the study was conducted according to the Declaration of Helsinki. Subjects received 10euro/h for participation.

In a first session, a standardized clinical interview [31] was conducted in order to make a clinical diagnosis of dental phobia and to screen for comorbid disorders in the phobia group. Exclusion criteria consisted of substance dependence (except nicotine), psychotic disorder, bipolar disorder, obsessive-compulsive disorder, posttraumatic stress disorder, major depressive disorder, blood phobia with fainting symptoms, intake of psychotropic medication, and any MRI contraindications. Six patients had a further diagnosis of another specific phobia (e.g. height phobia). For control participants exclusion criteria consisted of any diagnosis according to DSM-IV-TR.

During the second session, the subjects participated in an fMRI study, in which they were confronted with phobic, disgust, fear, and neutral pictures. The main results concerning the neural correlates of symptom provocation have been reported by [8].

The present study focused on the effects of individual differences in cognitive reappraisal usage on the neural correlates of habituation processes in dental phobics and healthy controls. For this purpose, participants completed the emotion regulation questionnaire (ERQ [20] German version [29]). This questionnaire is a 10-item measure to assess the habitual use of “cognitive reappraisal” and “expressive suppression” on a 7-point Likert scale (‘1’ = “strongly disagree” to ‘7’ = “strongly agree”). One example item for the cognitive reappraisal scale is for instance “When I want to feel less negative emotion, I change the way I’m thinking about the situation”. The mean score of the six reappraisal items was calculated for the purpose of the present study.

### Experimental design

During the fMRI session, participants were presented with 120 pictures representing the four emotional categories ‘Phobia’, ‘Fear’, ‘Disgust’, and ‘Neutral’ (30 pictures in each category). The pictures were taken from the International Affective Picture System (IAPS [32]) and from two other sets [33,34]. The phobic category consisted of pictures showing dental treatment (e.g. physician holding a dental drill in his hand). Fear stimuli depicted predators and attacks by humans, whereas disgust pictures showed e.g. repulsive animals and scenes from the domain ‘poor hygiene’. Neutral pictures consisted of household articles. During the experiment the pictures were randomly presented for 3 s each. The inter-stimulus intervals varied between 3 and 6 s and participants were presented with a white fixation cross on a black background. In addition, 30 null events (fixation cross) with a mean duration of 6s were presented throughout the experiment. After the experiment, subjects gave valence, arousal, fear and disgust ratings (9-point Likert scales) for each picture category block-wise.

### Data acquisition and analysis

Magnetic resonance imaging was carried out with a 1.5 Tesla whole-body tomograph (Siemens Symphony with a quantum gradient system) with a standard head coil. A total of 530 volumes was registered using a T2*-weighted gradient echo-planar imaging sequence (EPI) with 30 slices covering the whole brain (slice thickness = 4 mm; 1 mm gap; descending slice order; TA = 100 ms; TE = 55 ms; TR = 3 s; flip angle = 90°; field of view = 192 mm × 192 mm; matrix size = 64 pixel × 64 pixel; 3×3×4 mm voxel size). Due to an incomplete steady state of magnetization, the first three volumes were discarded. The orientation of the axial slices was parallel to the OFC tissue - bone transition. An anatomical T1-weighted scan (3D-MPRAGE, 1.4×1×1 mm voxel, FoV: 250 mm; TI: 1,100 ms, TE: 4.18 ms, TR: 1,990 ms, flip angle: 15°) was carried out to get highly resolved structural information for the normalization procedure and a gradient echo field map sequence to get information for unwarping B_0_ distortions (gre_fieldmap; 30 slices, 4 mm + 1 mm gap, in-plane: 3×3 mm, TE1: 10 ms, TE2: 14.76 ms, FoV: 192 mm, flip angle: 90°; comparable parameters to the echo-planar images).

Data analysis was done with the Statistical Parametric Mapping software (SPM8, Wellcome Department of Cognitive Neurology, London) implemented in MatLab R2007b (Mathworks Inc., Sherborn, MA). Unwarping and realignment, slice time correction, and normalization to the standard space of the Montreal Neurological Institute brain (MNI brain) were carried out. Smoothing was executed with an isotropic three dimensional Gaussian filter with a full width at half maximum (FWHM) of 9 mm. The four conditions were each modeled by one regressor and its time modulation (1^st^ order; models increasing activation across trials) in order to investigate the time course of brain activation. All regressors were modeled by a stick function convolved with the canonical hemodynamic response function (hrf) in the general linear model. The duration was set to 3 s for each event. Furthermore, the six movement parameters of the rigid body transformation applied by the realignment procedure were introduced as covariates in the model. A high-pass filter of 128 s was applied.

The contrasts ‘activation decrease (Phobia) vs. activation decrease (Neutral)’, ‘activation decrease (Phobia) vs. activation decrease (Fear)’ and ‘activation decrease (Phobia) vs. activation decrease (Disgust)’ were calculated on an individual level. One-sample *t*-tests were calculated for the analysis of habituation effects within the phobia group for these contrasts and two-sample *t*-tests for comparison of the phobic and the control group for the ‘activation decrease (Phobia) vs. activation decrease (Neutral)’ contrast. The correlation of cognitive reappraisal and symptom severity (Dental Anxiety Scale) with neural responses (activation decrease for phobic vs. neutral pictures) and of cognitive reappraisal with activation for phobic vs. neutral pictures was tested via *t*-contrasts in multiple regression analyses (second-level analyses), with the reappraisal or DAS score entered as the covariate of interest. Regions of interest (ROI) were the amygdala, insula, basal ganglia (pallidum, putamen, caudate nucleus), SMA, lateral orbitofrontal cortex (lOFC), dACC, rACC, dmPFC, vmPFC, dlPFC, and vlPFC. The significance threshold was set to α = 0.05 on voxel-level, corrected for multiple testing (family wise error (FWE) correction) in the respective search volume (ROI, whole brain) with an intensity threshold of *p* = .001 for ROI analyses. For exploratory whole brain analyses, an inclusive gray matter mask was used. ROI analyses were performed using the small volume correction option of SPM8. Amygdala and insula masks were maximum probability masks (probability threshold set to .50) taken from the current “Harvard-Oxford cortical and subcortical structural atlases” provided by the Harvard Center for Morphometric Analysis (http://www.cma.mgh.harvard.edu/), included in the FSL software package (http://www.fmrib.ox.ac.uk/fsl/). The dACC mask consisted of a 10 mm sphere surrounding a peak voxel for phobia specific neural responses in the anterior cingulate/mid-cingulate gyrus (MNI: x = 0, y = 0, z = 40) as indicated in a meta-analysis of symptom provocation studies in specific phobia [6]. The remaining masks were created by the MARINA software package [35].

## Results

### Questionnaire and rating data

For the cognitive reappraisal scale, the phobic patients reported a mean score of 4.75 (S*D* = 0.95; range: 2.17.-6.17) and for the Dental Anxiety Scale (DAS) a mean score of 17.37 (*SD* = 2.24; range: 11-20). There was no significant correlation of cognitive reappraisal with symptom severity as measured by the Dental Anxiety Scale in this group (*r* = .10, *p* = .62). The control group had a mean score of 4.65 on the reappraisal scale (*SD* = .70; range: 3.17-6.00) and for the DAS a mean score of 6.29 (*SD* = 1.65; range: 4-10), with no significant correlation between the reappraisal and the DAS score (*r* = -.19, *p* = .41). Additionally, there was no significant difference between the phobic group and the control group in dispositional cognitive reappraisal (*T*(46) = .39, *p* = .70).

The phobic group reported a mean score of *M* = 2.40 for valence (*SD* = 1.08), *M* = 6.00 for arousal (*SD* = 2.27), *M* = 6.15 for fear (*SD* = 1.94) and *M* = 3.89 for disgust ratings (*SD* = 2.31) of the phobic picture category. There was no significant association of cognitive reappraisal or DAS with valence, arousal, fear and disgust ratings of the phobic stimuli in the phobic group (all *p* > .2).

The control group reported a mean score of *M* = 5.63 for valence (*SD* = 1.57), *M* = 2.32 for arousal (*SD* = 1.49), *M* = 1.76 for fear (*SD* = 1.26) and *M* = 1.76 for disgust ratings (*SD* = 0.89) of the phobic picture category. Furthermore, there was no significant association of the cognitive reappraisal score with valence, arousal, fear and disgust ratings of the phobic stimuli in the control group (all *p* > .45). The same applies for the association of the DAS score with arousal, fear and disgust ratings (all p > .14), whereas the DAS was significantly (negatively) correlated with the valence ratings of the phobic pictures in the control group (*r* = -.503, *p* = .028).

### fMRI data

#### Activation decreases and increases

**Figure 1:**
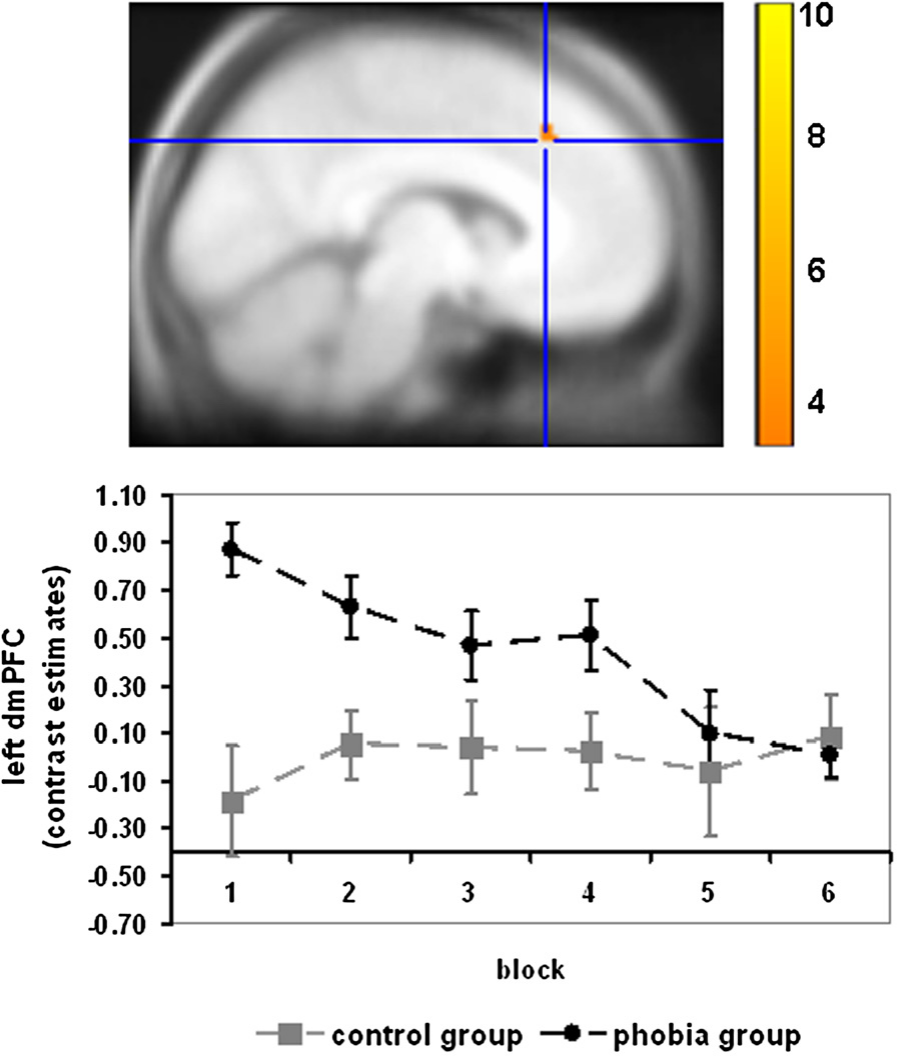
**Stronger activation decrease in the left dmPFC (MNI coordinate: -6, 29, 40; marginally significant: *p***_***fwe***_ **= .058) for phobic vs. neutral pictures in the phobia group compared with the control group.** For illustration purposes, contrast estimates were averaged across 5 consecutive trials, respectively, resulting in 6 blocks for 30 trials. Color bar indicates *T*-values; for illustration reasons, data were thresholded at *p* < .001 and superimposed on the MNI305-T1 template.

For phobic compared with neutral stimuli, several brain regions showed a decrease in activation over time in the phobic group. Especially the right middle temporal gyrus (exploratory analysis), right caudate nucleus, bilateral vmPFC, dmPFC, rACC, and left vlPFC showed a significant activation decline (see Table 1). Marginally significant responses were found for the left insula (*Z*_max_ = 3.30, *p* = .069), the left dlPFC (*Z*_max_ = 3.74, *p* = .071), and a further cluster in the right dmPFC (*Z*_max_ = 3.33, *p* = .098) and the left vlPFC (*Z*_max_ = 3.57, *p* = .071) (see Table 1). There were no activation increases for phobic compared with neutral pictures over time. Compared with the fear category, phobic patients showed a stronger activation decline during phobic stimulation in the insula (marginally significant; *Z*_max_ = 3.18, *p* = .097) and the left and (tendentially) right vlPFC (*Z*_max_ = 3.57, *p* = .076) (see Table 1). There were no stronger activation decreases for fear vs. phobic pictures. However, phobic compared with disgust pictures led to a stronger activation decrease in the right rACC, whereas less decrease for this contrast was observed in the right vmPFC (see Table 1). In order to investigate the specificity of the results we further conducted a comparison of the phobic and the healthy control group for phobic vs. neutral pictures. As the only result a (marginally significant) stronger activation decrease appeared in the left dmPFC (MNI coordinates: x = -6, y = 29, z = 40; *Z*_max_ = 3.54; *p*_fwe_ = .058) in phobic compared with control participants (see Figure 1).

**Table 1:** Neural activation decrease over time in the phobic group

**Structure**	**H**	**x**	**y**	**z**	**Z**_**max**_	**p**_**fwe**_
**Phobia gt neutral**						
Middle temporal gyrus	L	−45	14	−29	4.92	.029E
insula	L	−30	20	−8	3.30	.069^+^
caudate nucleus	R	21	−16	22	3.45	.037
dlPFC	L	−24	32	46	3.74	.071^+^
dmPFC	L	−6	29	40	4.79	.001
						.050E^+^
dmPFC	L	−3	53	7	3.99	.017
dmPFC	R	6	47	4	4.33	.004
dmPFC	R	6	32	43	3.33	.098^+^
rACC	L	−3	50	7	4.00	.006
rACC	R	6	44	4	3.87	.009
vlPFC	L	−39	29	13	4.14	.011
vlPFC	L	−45	17	13	3.57	.071^+^
vmPFC	L	−3	56	−11	4.05	.008
vmPFC	R	9	59	−14	3.76	.021
**Neutral gt phobia**						
*no significant results*						
**Phobia gt fear**						
insula	L	−39	−1	4	3.18	.097^+^
vlPFC	L	−45	8	10	3.94	.023
vlPFC	R	48	17	1	3.57	.076^+^
**Fear gt phobia**						
*no significant results*						
**Phobia gt disgust**						
rACC	R	6	38	13	3.94	.031
**Disgust gt phobia**						
vmPFC	R	9	29	−23	3.57	.034
vmPFC	R	3	23	−23	3.52	.039

Neural activation decrease over the course of symptom provocation for phobic compared with neutral pictures for exploratory whole brain analyses (marked with ^E^) and regions of interest analyses (p_fwe_ < .05); tendentially significant results p_fwe_ < .1 are marked with ^+^; all coordinates (*x, y, z*) are given in MNI space. *H*, hemisphere; *L*, left *R*, right; *Z*_*max*_, maximum Z-value *p*_*fwe*_, fwe-corrected p-value (small-volume correction).

#### Correlations with dispositional cognitive reappraisal and symptom severity

**Figure 2:**
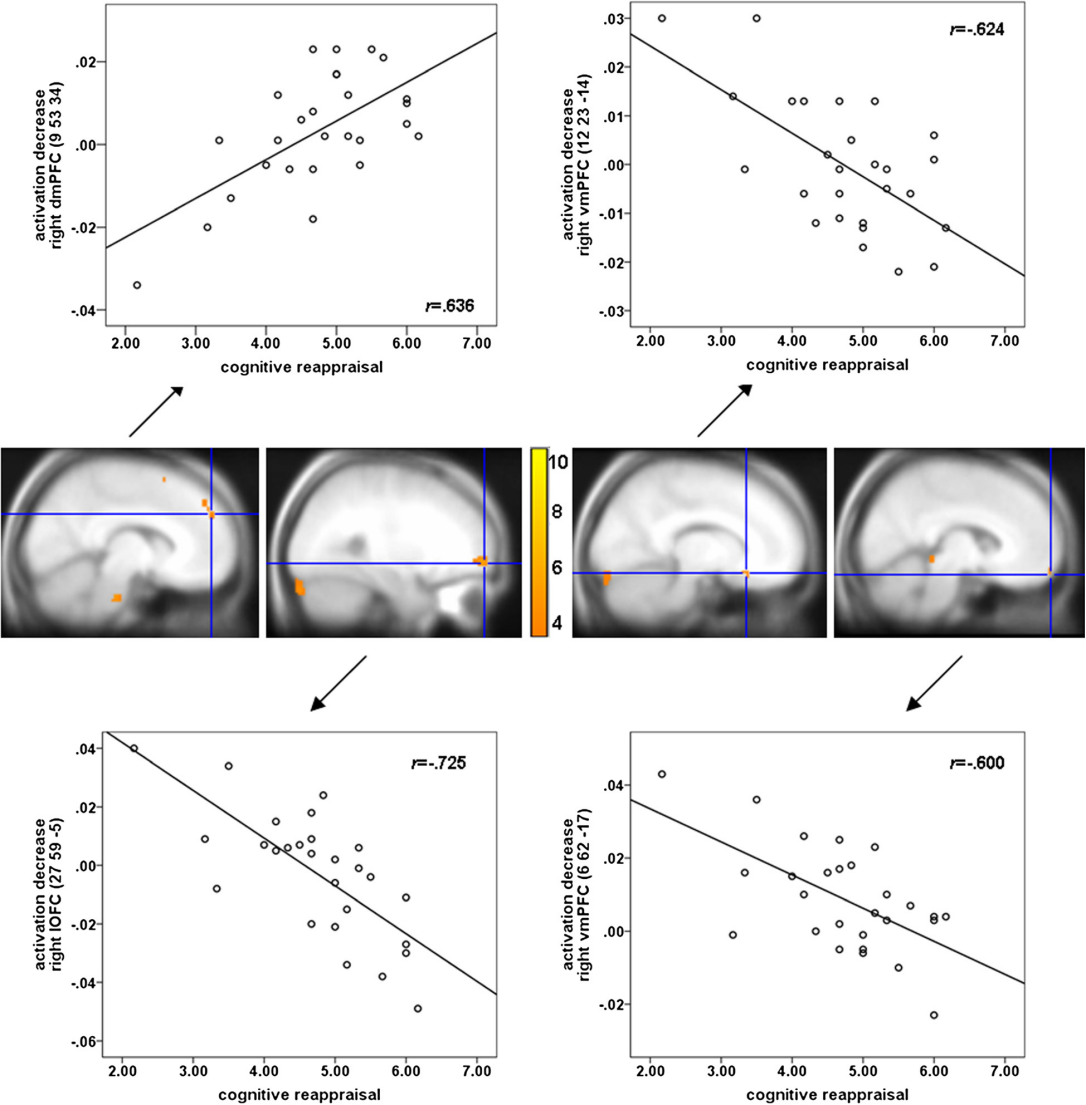
**Positive association of dispositional cognitive reappraisal with activation decrease in the right dmPFC (MNI coordinate: 9, 53, 34; marginally significant: *p***_***fwe***_ ***=*** **.052) in the phobic group; negative associations of dispositional cognitive reappraisal with activation decrease in the right lOFC (MNI coordinate: 27, 59, -5; *p***_***fwe***_ **= .008), the right vmPFC (MNI coordinate: 12, 23, -14; marginally significant: *p***_***fwe***_ **= .053), and a further cluster in the right vmPFC (MNI coordinate: 6, 62, -17; marginally significant: *p***_***fwe***_ **= .076) in the phobic group.** Color bar indicates *T*-values; for illustration reasons, data were thresholded at *p* < .001 and superimposed on the MNI305-T1 template.

In the phobic group, dispositional cognitive reappraisal was correlated with a stronger activation decrease in the right dmPFC (marginally significant; *Z*_max_ = 3.56, *p* = .052) for phobic compared with neutral pictures (see Table 2a and Figure 2). A negative correlation of cognitive reappraisal with activation decreases for phobic vs. neutral pictures was observed for the right lOFC and two clusters in the right vmPFC (marginally significant; *Z*_max_ = 3.45, *p* = .053 and *Z*_max_ = 3.33, *p* = .076) (see Table 2a and Figure 2). Furthermore, the correlation of symptom severity with activation decreases in response to phobic vs. neutral stimuli in the phobic group yielded no significant results.

**Table 2:** Correlation of dispositional cognitive reappraisal with neural activation decrease for phobic vs. neutral pictures

**Structure**	**H**	**x**	**y**	**z**	**Z**_**max**_	**p**_**fwe**_
**a) Phobic group**						
**positive correlation with dispositional cognitive reappraisal**						
dmPFC	R	9	53	34	3.56	.052^+^
**negative correlation with dispositional cognitive reappraisal**						
lOFC	R	27	59	−5	4.27	.008
vmPFC	R	12	23	−14	3.45	.053^+^
vmPFC	R	6	62	−17	3.33	.076^+^
**b) Control group**						
**positive correlation with dispositional cognitive reappraisal**						
dlPFC	L	−39	26	43	3.99	.031
SMA	L	−3	8	43	3.38	.074^+^
SMA	R	6	−16	73	3.43	.069^+^
vlPFC	L	−45	17	7	4.46	.055^+^
**negative correlation with dispositional cognitive reappraisal**						
*no significant results*						

Correlation of dispositional cognitive reappraisal with neural activation decrease over the course of symptom provocation for phobic compared with neutral pictures in the a) phobic and b) control group for exploratory whole brain analyses (marked with ^E^) and regions of interest analyses (p_fwe_ < .05); tendentially significant results p_fwe_ < .1 are marked with ^+^; all coordinates (*x, y, z*) are given in *MNI* space. *H* hemisphere, *L* left, *R* right, *Z*_*max*_ maximum Z-value, *p*_*fwe*_ fwe-corrected p-value (small-volume correction).

In order to assess the generalizability of the results, a correlation of dispositional cognitive reappraisal and activation decreases was calculated within the control group. Hereby, we observed a positive correlation of dispositional cognitive reappraisal with activation decreases in the left dlPFC and marginally significant with the SMA (left: *Z*_max_ = 3.38, *p* = .074; right: *Z*_max_ = 3.43, *p* = .069) and left vlPFC (*Z*_max_ = 4.46, *p* = .055) (see Table 2b).

For comparability with previous studies, we further conducted a correlation of dispositional cognitive reappraisal with neural activation in response to phobic compared with neutral stimuli. In the phobic group, we observed a negative correlation with cognitive reappraisal in the left SMA, the right vlPFC, bilateral dmPFC and marginally significant in the left dlPFC (*Z*_max_ = 3.55, *p* = .091), lOFC (*Z*_max_ = 3.56, *p* = .056), rACC (*Z*_max_ = 3.10, *p* = .074) and two clusters in the vlPFC (*Z*_max_ = 3.57, *p* = .051 and *Z*_max_ = 3.43, *p* = .075) (see Table 3a). No positive correlation with dispositional cognitive reappraisal was found for this group. The control group showed a positive correlation of reappraisal with the left amygdala and the left insula as well as marginally significant with right insula (*Z*_max_ = 3.37, *p* = .060) and left lOFC (*Z*_max_ = 3.70, *p* = .055) activation (see Table 3b).

**Table 3:** Correlation of dispositional cognitive reappraisal with neural activation for phobic vs. neutral pictures

**Structure**	**H**	**x**	**y**	**z**	**Z**_**max**_	**p**_**fwe**_
**a) Phobic group**						
**positive correlation with dispositional cognitive reappraisal**						
*no significant results*						
**negative correlation with dispositional cognitive reappraisal**						
dlPFC	L	−42	8	46	3.55	.091^+^
SMA	L	−12	14	64	3.93	.011
dmPFC	L	−9	53	25	3.75	.027
dmPFC	R	12	29	52	3.59	.035
lOFC	L	−54	23	−8	3.56	.056^+^
rACC	L	−9	38	10	3.10	.074^+^
vlPFC	L	−33	5	28	3.57	.050^+^
vlPFC	L	−45	29	13	3.57	.051^+^
vlPFC	L	−48	20	25	3.43	.075^+^
vlPFC	R	51	17	31	4.05	.011
**b) Control group**						
**positive correlation with dispositional cognitive reappraisal**						
insula	L	−39	2	−14	3.69	.024
insula	R	39	16	10	3.37	.060^+^
amygdala	L	−30	−1	−23	3.14	.035
lOFC	L	−27	29	−8	3.70	.055^+^
**negative correlation with dispositional cognitive reappraisal**						
*no significant results*						

Correlation of dispositional cognitive reappraisal with neural activation for phobic compared with neutral pictures in the a) phobic and b) control group for exploratory whole brain analyses (marked with ^E^) and regions of interest analyses (p_fwe_ < .05); tendentially significant results p_fwe_ < .1 are marked with ^+^; all coordinates (*x, y, z*) are given in *MNI* space. *H* hemisphere, *L* left, *R* right, *Z*_*max*_ maximum, *Z-value p*_*fwe*_, fwe-corrected p-value (small-volume correction).

## Discussion

The aim of this fMRI study was to investigate the association of dispositional cognitive reappraisal with neural temporal dynamics during phobic stimulation in dental phobia. For this purpose, the time course of brain activation during symptom provocation with visual phobic stimuli was investigated in a sample of 27 dental phobic patients and 21 healthy controls. The habitual use of cognitive reappraisal was assessed with the Emotion Regulation Questionnaire (ERQ [20] German version [29]) in order to investigate the association of dispositional reappraisal with activation decreases over the course of symptom provocation.

A general decrease of activation for phobic compared with neutral stimuli was observed in several brain regions including the insula (marginally significant), the middle temporal gyrus, and the caudate nucleus. Some of these regions have previously been shown to be overactive during symptom provocation in specific phobia [6,8] and might therefore be associated with reduced emotional responding respectively stronger habituation over time in the present study. Concerning the specificity of the results, our data mainly demonstrate that the activation decrease in most of the reported brain regions does not differ from the time course of activation in response to fear and disgust pictures as well as when comparing phobic patients with healthy control subjects. However, a stronger activation decrease in the insula (marginally significant) was also observed in response to phobic compared with fear pictures. As mentioned above, several studies reported a hyperactivation of the insula during symptom provocation in specific phobia (for an overview see [6]). Moreover, the insula is a central structure in interoceptive perception and awareness [36]. One study has even demonstrated an anatomical overlap of insula activation during interoception and symptom provocation in patients with specific phobia [37]. Stronger activation decrease in the insula might therefore be associated with enhanced habituation of bodily arousal in response to phobic compared with fear stimuli, probably due to heightened responding in the insula during early confrontation with phobic stimuli. Habituation of insula and middle temporal gyrus activation, as found in our study, has already been demonstrated in spider phobic subjects [15]. However, it needs to be emphasized that activation decrease in the insula was only marginally significant and needs to be interpreted with caution. Further studies are needed to replicate the current results.

In addition, several regulation-related prefrontal and anterior cingulate cortex areas showed a reduction of activation over the time course of the experiment. Stronger habituation was found in the vlPFC even for phobic compared with fear pictures and in the rACC for phobic compared with disgust pictures. This might be interpreted as a reduction of cognitive control processes over the course of symptom provocation. On the one hand, this might be due to a reduced need for cognitive control based on a decline of emotional responding, and thus might be adaptive. On the other hand, the activation decrease might be associated with reduced (although necessary) cognitive control or extinction processes, and thus might be non-adaptive. This might be more pronounced in response to phobic stimuli in the vlPFC and the rACC due to a higher salience of phobic stimuli that require stronger cognitive processing and regulation especially in the beginning of confrontation.

The main goal of the present study was to investigate the association of dispositional cognitive reappraisal with neural temporal dynamics during processing of phobic stimuli in dental phobia.

As a main result a stronger activation decrease in the right dmPFC (marginally significant) was found in frequent reappraisers in the phobia group. This could possibly be explained by diminished cognitive regulation of negative emotions over time. Additionally, an enhanced activation decrease in the left dmPFC over the course of symptom provocation was also observed in phobic compared with healthy control subjects in the present study and points further to the important role of this region. The dmPFC is crucially involved in emotion regulation processes via cognitive reappraisal (for an overview see [21]). Additionally, previously observed reduced dmPFC activation during phobia-specific cognitive reappraisal points to an important role of this region in phobia-specific cognitive emotion regulation [12]. A stronger activation decrease over time might be related to a stronger decline of explicit cognitive emotion regulation over the course of the experiment. This might be due to a reduced need to effortfully down-regulate negative emotions because of a more effective automatic regulation or reduced emotional responding.

In line with this interpretation, dispositional reappraisal has been shown to be associated with a diminished activation decrease in the vmPFC over time in the present study. The vmPFC has previously been found to exhibit reduced activation during symptom provocation in patients with specific phobia [11-13]. Moreover, reduced activation of the vmPFC along with amygdala hyperactivation has been observed during the acquisition of conditioned fear responses [38] most likely indicating reduced cognitive control of emotional reactions. In addition, vmPFC activation has previously been found during extinction learning and recall [38] as well as a result of successful CBT in specific phobia [13]. Regarding these results, the observed reduced decrease of vmPFC activation in the present study in individuals more frequently using cognitive reappraisal might be related to a decreased fear recall or stronger extinction learning over the course of symptom provocation. This interpretation fits well with the observed reduced activation decrease in the vmPFC in response to phobic compared with disgust stimuli in phobic subjects.

In addition, lateral OFC activation showed a diminished decline in individuals with a higher habitual use of cognitive reappraisal. This region has frequently been found to be activated during symptom provocation in specific phobia [39,40] and seems to be especially involved in the processing of negative affective states [41]. Hence, the association of dispositional reappraisal with sustained lOFC activation during symptom provocation might be related to prolonged negative affective processing. This interpretation seems to contradict the hypothesis of stronger extinction learning in individuals high in dispositional cognitive reappraisal. However, the OFC has also been found to play a crucial role in the regulation of emotions via cognitive reappraisal (for an overview see [21]). Therefore, OFC activation might more likely reflect the activation and reinterpretation of negative appraisals in response to phobic stimuli, which is more sustained in subjects high in dispositional reappraisal over the course of symptom provocation.

The results for the correlation of cognitive reappraisal with activation decreases for phobic vs. neutral pictures in control subjects differ from the results of the phobia group. In the control group, a stronger activation decrease for frequent reappraisers was observed in the dlPFC, vlPFC and SMA and might be interpreted as a reduced employment of cognitive control strategies over the course of the experiment. The differences to the phobia group, however, indicate that there might be differences in the underlying processes. On the one hand, phobic and control subjects differ in the extent of emotional responding towards phobic stimuli which might result in different correlations with dispositional cognitive reappraisal. On the other hand phobics and controls might use different tactics of cognitive reappraisal (i.e. distancing vs. reinterpretation) leading to distinct activation patterns. Further studies are needed in order to disentangle the underlying mechanism by investigating for instance instructed rather than dispositional cognitive reappraisal.

Additional analyses focused on the association of dispositional cognitive reappraisal and activation rather than on the activation decrease during symptom provocation. A number of regulation-related prefrontal, anterior cingulate and orbitofrontal cortex areas were found to be negatively correlated with cognitive reappraisal in the phobic group. This supports the hypothesis that individuals with higher reappraisal abilities do not need to recruit these regulation-related brain areas as much as individuals with lower reappraisal abilities to control their emotions during symptom provocation. Enhanced insula, amygdala and lOFC responses in frequent reappraisers in the control group indicate, however, a more pronounced emotional processing in these individuals. This might probably derive from a stronger engagement with emotional aspects of the stimuli during reappraisal and needs to be further investigated in future studies.

Because there was no association of cognitive reappraisal and symptom severity as well as symptom severity and time course of brain activation one might speculate that cognitive reappraisal is a better predictor of habituation or even emotional relearning during exposure than symptom severity. Therefore, it might be of special importance to assess the general cognitive reappraisal abilities of phobic patients prior to exposure sessions and to improve these abilities if necessary in order to strengthen the (long-term) outcome of CBT.

## Conclusions

In conclusion, the results of this study indicate that individual differences in cognitive reappraisal usage differentially modulate the time course of brain activation during symptom provocation in distinct brain areas. A stronger decline of dmPFC activation might be related to a diminished (need for) explicit emotion regulation over the course of symptom provocation. This result is in line with a diminished habituation of the vmPFC and the lOFC, regions important in more automatic emotion regulation processes like extinction learning. The vmPFC has even been shown to be associated with successful CBT in spider phobic patients [13]. Individuals high in dispositional cognitive reappraisal might be faster or even better at regulating their emotional responses with less effort, leading to a more successful emotional relearning. Due to the assumed stronger habituation and probably enhanced extinction learning, one could speculate that individuals high in dispositional cognitive reappraisal might be more able to benefit from CBT interventions. Or the other way round, strengthening cognitive reappraisal abilities in individuals low in dispositional cognitive reappraisal might be a promising way to enhance therapy success for a larger number of individuals in the short- and the long-term.

### Limitations

Some limitations of our study need to be mentioned. As we used a correlational approach, it is not clear if dispositional cognitive reappraisal leads to stronger habituation/extinction or if individual differences in these habituation/extinction processes preceded and influenced the habitual use of cognitive reappraisal. In addition, the present study investigated processes taking place during one single exposure session. In order to evaluate the association of individual differences in cognitive reappraisal with long-lasting neural changes, the recall of the extinction memory needs to be studied. Future studies investigating cognitive reappraisal and the neural correlates of extinction processes in specific phobia over the course of ‘real’ exposure therapy are important further steps in order to validate the current findings and optimize existing psychological interventions in specific phobia.

## Competing interests

All authors declare no financial or non-financial competing interests.

## Authors’ contributions

AS and AH designed this study. VL and WS conducted diagnostics and data acquisition. AH conducted the statistical analyses and AH, DV and RS interpreted the data. AH drafted the manuscript. DV and RS edited and revised the manuscript. All authors read and approved the final manuscript.
